# Quality of sleep and excessive daytime sleepiness among medical students in a Brazilian private university

**DOI:** 10.1590/1806-9282.20231141

**Published:** 2024-04-22

**Authors:** Anna Karolinna Ribeiro Souza, Rhaissa Siqueira Sandes, Raquel Fernandes Vanderlei Vasco, Edoarda Vasco de Albuquerque Albuquerque

**Affiliations:** 1 Centro Universitário de Maceió (UNIMA/Afya) – Maceió (AL), Brazil.

**Keywords:** Sleep hygiene, Students, medical, Sleepiness

## Abstract

**OBJECTIVE:**

The aim of this study was to assess medical students’ quality of sleep and excessive daytime sleepiness in different graduation cycles.

**METHODS:**

A cross-sectional study was carried out at a private university in Maceió, Brazil, from August 2021 to March 2022. The sample was composed of medical students aged 18 years and above from years 1–2 (basic cycle), 3–4 (clinical cycle), and 5–6 (internship) of Medical School who were invited to answer two validated questionnaires: the Pittsburgh Sleeping Quality Index and the Epworth Sleepiness Scale.

**RESULTS:**

A total of 179 students participated; most of them were female (78.2%), aged 19–25 years (73.7%), and with a body mass index<25 kg/m^2^ (73.7%), with smaller participation from students from the basic cycle (21.2%). Analyzing the Pittsburgh Sleeping Quality Index, 55.9% of the students were classified as having poor sleep quality, with no difference in sleep category between gender, age, body mass index, and graduation cycle. Students with a body mass index of ≥25 kg/m^2^ had longer sleep latency (p=0.016) and shorter sleep duration (p=0.027). The Epworth Sleepiness Scale assessment showed that 44.1% of the students exhibit daytime sleepiness. Women had more daytime sleepiness than men (p=0.017), with no difference between age, body mass index, and graduation cycle.

**CONCLUSION:**

About half of the medical students experience poor sleep quality and daytime sleepiness, regardless of the graduation cycle. This should trigger a targeted institutional intervention to promote better mental and physical health, as well as sleep hygiene, to reduce future health issues.

## INTRODUCTION

Sleep disorders, mainly insomnia and excessive daytime sleepiness (EDS), are a public health issue^1^. Prolonged sleep deprivation can be deleterious to physical and cognitive performance since higher functions, such as learning and memory retention, and also metabolic health, depend on sleep quality^2–4^.

Medical students are often subjected to an extensive curriculum, exhausting extracurricular activities, and internships with night shifts, and all of that combined can modify sleep architecture^5–9^. Evidence shows that poor sleep quality can lead to lower academic performance, compromising students’ abilities to reason, acquire, and process information, correctly interpret and solve clinical cases, and make students more prone to depression and anxiety^6,8,9^. In addition, sleep deprivation also increases reaction time and systolic blood pressure post-exercise, and the first could magnify the risk of accidents, both work-related and not work-related^6,10^.

All of these could generate a vicious cycle where students deprive even more of their sleep time to study for exams and increase substance abuse (caffeine and/or medications)^5,11^. This creates a chronic problem and is often overlooked by professors and other medical professionals, labeling this situation as "normal" and, in a certain way, promoting sleep deprivation^11^.

In this setting, considering the population studied as a risk factor for developing sleep disorders and knowing that there is an interference of these disorders over academic performance^8^, this study proposed to assess sleep quality and EDS among medical students from a private university in the Brazilian Northeast, comparing these aspects in different graduation cycles.

## METHODS

### Study design and participants

An observational, descriptive cross-sectional study was conducted at Centro Universitário Tiradentes (UNIT-AL), a private university in Maceió, Alagoas, from August 2021 to March 2022. The study population included medical students from years 1 to 6, allocated into three groups: basic cycle (BC; years 1–2), clinical cycle (CC; years 3–4), and internship (IN; years 5–6). During this study, the university had 864 registered medical students, distributed into 302 students in BC, 302 students in CC, and 260 in IN. The questionnaires were answered online, using the tool Google Forms to collect the data. Students from all years were recruited by online invitation during the pandemic and also by personal approach when the flexibilization of social distance measures was possible, providing a quick response code to the questionnaires for the participants.

The inclusion criteria were students aged ≥18 years registered in the institution's medical school. Those who filled out the forms incorrectly or incompletely were excluded. This study was approved by the local Ethics Committee (CAAE 48301921.3.0000.5641).

### Instruments

The selected instruments were the Pittsburgh Sleep Quality Index (PSQI), in its translated and validated version to Brazilian Portuguese^12^, and the Epworth Sleepiness Scale (ESS), also validated for usage in Brazil^13^. Demographic data regarding gender, age, body mass index (BMI), and previous sleep or chronic disease diagnoses were also collected.

Pittsburgh Sleeping Quality Index assesses sleep quality and disturbances in the last month, and it is composed of 19 items—and five more for individuals who share the bedroom or bed with another individual. These items are arranged in seven components: (1) subjective sleep quality, (2) sleep latency, (3) sleep duration, (4) habitual sleep efficiency, (5) sleep disturbances, (6) use of sleeping medication, and (7) daytime dysfunction. The global score achieved ranges from 0 to 21 points, and scores from 0 to 5 indicate good sleep quality, scores from 6 to 10 indicate poor sleep quality, and >10 points may indicate a sleep disorder.

ESS is a tool to identify excessive daytime sleepiness, including eight items that grade the probability of the individual dozing in inappropriate situations. The scoring system is from 0 to 3, in which 0 means "would never doze," 1 means "slight chance of dozing," 2 means "moderate chance of dozing," and 3 means "high chance of dozing." The global score ranges from 0 to 24. Scores >10 points suggest EDS, and scores >15 indicate severe daytime sleepiness.

### Statistical analysis

Statistical analysis was performed in IBM SPSS Statistics Client version 22.0 Multilingual, with a confidence level of 95% (p<0.05). Sample calculation was performed in G*Power version 3.1.7 using goodness-to-fit multiple proportions with an α-error of 0.05, power of 0.8, and effect size ω of 0.3, resulting in a total of 108 subjects, with 36 in each group. Individuals were allocated into groups related primarily to graduation cycle (BC, CC, and IN), as well as to gender (male and female), age (19–25 and 26 years or older), and BMI (<25 kg/m² and ≥25 kg/m²). We used the Shapiro–Wilke test to assess the variable distribution and the Levene test to evaluate homogeneity. We used the t-test to compare two parametric variables and the analysis of variance to compare three variables. For comparison of non-parametric variables, we used the Mann–Whitney test when two non-paired variables were analyzed and the Kruskal–Wallis test when three or more non-paired and non-homogeneous variables were analyzed.

## RESULTS

A total of 179 students answered both questionnaires (20.7% of the total) from all graduation cycles (BC=38, 21.2%; CC=72, 40.2%; IN=69, 38.5%). Most participants were female (n=140, 78.2%), aged 19–25 years (n=132, 73.7%), with a BMI<25 kg/m² (n=132, 73.7%). Around 23.7% of women and 44.7% of men were overweight or obese (p=0.018), and there was no significant difference between age and gender, or BMI and gender. Twenty-four students (13.4%) reported chronic diseases such as hypothyroidism (n=5) and asthma (n=4). Nine participants (5.0%) reported previous sleep disorder diagnoses, notably insomnia (n=4).

Regarding sleep disturbances in the last month, at least once a week, 56.9% (n=102) of the participants could not sleep within 30 min, 58.1% (n=104) woke up in the middle of the night, 50.2% (n=90) had to get up to use the bathroom, 16.2% could not breathe comfortably, 17.3% (n=31) coughed or snored loudly, 39.6% had bad dreams, 15.1% (n=27) had pain, and 16.7% (n=30) used prescribed or "over the counter" medicine to help sleep. Moreover, 39.1% (n=70) had trouble staying awake while driving, eating meals, or during social activities at least once a week, and 83.4% (n=149) reported not having enough enthusiasm to perform daily activities.

About 29.6% (n=53) of the participants shared a bed or bedroom with another individual. Their partners reported that at least once a week, 13.2% (n=7) of them snored loudly, 33.9% (n=18) twitched their legs or jerked, and 13.2% had episodes of disorientation or confusion during sleep.

Concerning the reported sleep/waking times, students go to sleep around 11:35 p.m., with a median sleep latency of 20 min, and wake up around 06:38 a.m., with a median sleep duration of 6.5 h (Table 1). Sleep duration among individuals aged 19–25 years was longer than among individuals aged ≥26 years, and sleep duration was also longer and sleep latency was shorter in individuals whose BMI was <25 kg/m² than whose BMI was ≥25 kg/m² (Table 1).

**Table 1:** Sleep latency and duration according to graduation cycle, gender, age, and body mass index.

		Sleep latency (IQR)	p	Sleep duration (IQR)	p-value
Total		20 (10–30)		6.5 (5.5–7.0)	
**Gender**					
	Male	20 (10–30)	0.789	6.5 (5.5–7.0)	0.656
	Female	20 (10–30)		6.0 (6.0–7.0)	
**Age (years)**					
	19–25	20 (10–30)	0.054	7.0 (6.0–7.0)	**0.025**
	>25	30 (15–40)		6.0 (5.5–7.0)	
**BMI (kg/m^2^)**					
	<25	20 (10–30)	**0.016**	7.0 (6.0–7.3)	**0.027**
	≥25	30 (15–40)		6.0 (5.5–7.0)	
**Graduation cycles**					
	Basic	20 (10–41.2)	0.824	6.0 (5.5–7.0)	0.208
	Clinical	20 (10–30)		7.0 (6.0–7.0)	
	Internship	25 (10–32.5)		6.5 (5.5–7.5)	

Statistically significant values are denoted in bold.

**Figure 1:**
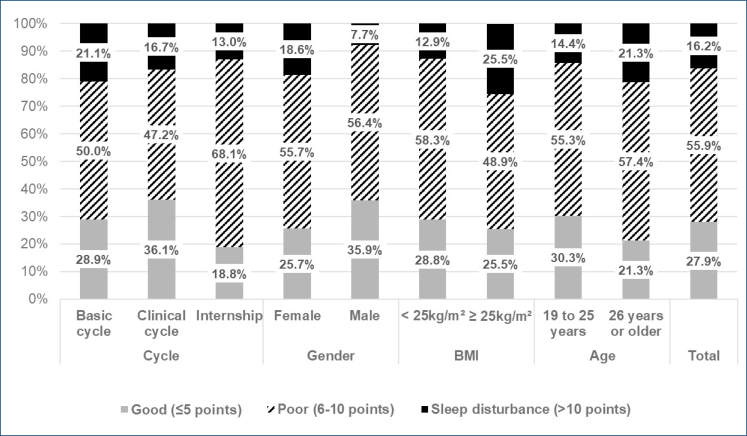
Distribution according to sleep quality classification by Pittsburgh Sleep Quality Index among graduation cycles, gender, body mass index, and age. Note: there was no statistically significant difference among any of the groups.

The median PSQI score was 7 points [interquartile range (IQR): 5–10 points]. In a categorical analysis, 55.9% of the participants obtained 5–10 points, therefore being classified as having poor sleep quality (Figure 1). There was no significant difference in the PSQI among the graduation cycles (p=0.09, Figure 1) or scores among various PSQI components. Nevertheless, in component 1, which assesses the subjective quality of sleep, 34.2% of participants from BC, 20.8% from CC, and 14.5% from IN classified their sleep quality as poor in the last month (Figure 1).

Regarding gender, there was no difference in the assortment of categories among men and women (p=0.186, Figure 1). In a subanalysis of the various components, women had higher scores in components 5, sleep disturbances (2 versus 1 point, p=0.01), and 7, daytime dysfunction (2 versus 1 point, p=0.024). There was no difference between age groups in category assortment or individual scale components by PSQI (Figure 1, Table 1). Concerning BMI, there was no difference among category assortments (p=0.128, Figure 1).

Analyzing ESS, 44.1% (n=79) of the sample showed some degree of daytime sleepiness. The mean score was 9.8±4.2, and women had a worse score (female 10.4±4.2; male 7.5±3.3; p<0.001) and were more assorted as having EDS (Table 2). Younger individuals had higher scores (19–25 years 10.3±4.0; ≥26 years 8.3±4.5; p=0.004), but they did not differ in daytime sleepiness classification (Table 2).

**Table 2:** Daytime sleepiness classification according to Epworth Sleepiness Scale distributed by graduation cycle, gender, age, and body mass index.

		No EDS (<10 points)	EDS (10–15 points)	Severe EDS (>15 points)	p-value
Total		55.9%	33.0%	11.2%	
**Gender**					
	Male	71.8%	28.2%	0.0%	**0.017**
	Female	51.4%	34.3%	14.3%	
**Age (years)**					
	19–25	51.5%	36.4%	12.1%	0.144
	>25	68.1%	23.4%	8.5%	
**BMI (kg/m^2^)**					
	<25	53.0%	34.8%	12.1%	0.434
	≥25	63.8%	27.7%	8.5%	
**Graduation cycles**					
	Basic	44.7%	44.7%	10.5%	0.149
	Clinical	66.7%	23.6%	9.7%	
	Internship	50.7%	36.2%	13.0%	

Statistically significant values are denoted in bold. EDS: excessive daytime sleepiness.

## DISCUSSION

In this study, medical students reported a median sleep duration of 6.5 h. Although total sleep time may vary according to age, the mean value is around 7–8 h per night^14^. Therefore, it is noticeable that our sample has less sleep time than recommended, and individuals with less than 7 h of sleep are more likely to develop health issues, regardless of gender^14^.

Likewise, 55.9% of medical students had poor sleep quality, and 16.2% had a probable sleep disturbance. In a study performed on the adult population also from Maceió, poor sleep quality was found in 48% of participants^15^, reinforcing the idea that medical students are at risk of sleep disturbances^7^. In a study performed in Botucatu, 87.1% of medical students obtained >5 points in PSQI, a rate higher than our study (72.1%) and the 61.5% were found in Paraiba, Brazil^16,17^. In parallel, a study assessing bruxism and smartphone usage with the performance of PSQI in Brazil showed a median score of 7 points, similar to our findings^18^. Regarding daytime sleepiness, about 11.6–36% of the general population exhibited EDS^19^, while in our study this rate was higher at 44.1%. Daytime sleepiness directly impacts performance, and 83.4% of the students felt they lacked enthusiasm for daily activities. This could lead to impaired learning and memory, thus increasing psychological distress in this population^20^. Curiously, women showed more daytime sleepiness than men, which could be a selection bias since male participation was low.

The participation of students from BC was 12.5% of the registered students in this cycle, lower than the other two assessed periods (CC 23.8% and IN 26.5%). A possible explanation is the selected age of 18 years or older for participation in this study since first-year students have a higher proportion of underaged individuals. Nevertheless, the participation in the BC group was enough to achieve the minimum number of subjects necessary to maintain this study's strength.

Although about half of the students were classified as poor sleepers, analyzing component 1 of PSQI, only 34.2% of the participants from BC, 20.8% from CC, and 14.5% from IN agreed with this classification, showing a different perception of the students from what was measured by PSQI. This emphasizes that sleep quality and self-consciousness about lifestyle need debating during graduation. Also, 16.7% of the population used hypnotic medications in the last month, a higher rate compared to similar studies in other universities around Brazil, where the mean rate of medication usage was 8.6%^16,19^.

Regarding weight, individuals with a BMI≥25 kg/m² had higher PSQI scores, shorter sleep duration, and longer sleep latency. There is a correlation between obesity and poor sleep quality, often related to sleep apnea. However, a study in Kuwait with a cohort of individuals without sleep apnea observed an independent effect of weight on sleep quality, and BMI values were directly proportional to worse PSQI scores and shorter sleep duration and efficiency^21^.

Concerning the graduation cycles, despite no statistical significance, internship students exhibit a worse quality of sleep, followed by the basic and then clinical cycles, without difference in daytime sleepiness, a similar pattern found in the countryside of Paraíba, Brazil^17^. This corroborates that there is a reduction in available time for adequate sleep over the graduation years, probably due to workload and night shifts. The finding of worse quality sleep in basic compared with the clinical cycle can be justified by the transition from high school to university^14,16^.

This study has several limitations. Overall participation of students from BC was low, probably due to the cutoff age of 18 years, which could attenuate differences among graduation cycles due to selection bias. Furthermore, male participation and individuals with a BMI≥25 kg/m² were also low, and the findings may not be generalized to these populations.

## CONCLUSION

In this study, we identified a high prevalence of poor sleep quality and EDS among medical students from a private university. These findings should trigger an intervention to promote better mental and physical health, along with sleep hygiene, to reduce future health issues.
